# *E. coli* Nissle 1917 Affects *Salmonella* Adhesion to Porcine Intestinal Epithelial Cells

**DOI:** 10.1371/journal.pone.0014712

**Published:** 2011-02-17

**Authors:** Peter Schierack, Sylvia Kleta, Karsten Tedin, Julius Tachu Babila, Sibylle Oswald, Tobias A. Oelschlaeger, Rico Hiemann, Susanne Paetzold, Lothar H. Wieler

**Affiliations:** 1 Institut für Mikrobiologie und Tierseuchen, Freie Universität Berlin, Berlin, Germany; 2 Fachbereich Bio-, Chemie- und Verfahrenstechnik, Hochschule Lausitz (FH), Senftenberg, Germany; 3 Bundesinstitut für Risikobewertung, Berlin, Germany; 4 Department of Neuroproteomics, Max Delbrueck Center for Molecular Medicine, Berlin, Germany; 5 Institut für Molekulare Infektionsbiologie, Universität Würzburg, Würzburg, Germany; 6 Max Planck Institut für Infektionsbiologie, Berlin, Germany; 7 Research Center Borstel, Borstel, Germany; Universita di Sassari, Italy

## Abstract

**Background:**

The probiotic *Escherichia coli* strain Nissle 1917 (EcN) has been shown to interfere in a human *in vitro* model with the invasion of several bacterial pathogens into epithelial cells, but the underlying molecular mechanisms are not known.

**Methodology/Principal Findings:**

In this study, we investigated the inhibitory effects of EcN on *Salmonella Typhimurium* invasion of porcine intestinal epithelial cells, focusing on EcN effects on the various stages of *Salmonella* infection including intracellular and extracellular *Salmonella* growth rates, virulence gene regulation, and adhesion. We show that EcN affects the initial *Salmonella* invasion steps by modulating *Salmonella* virulence gene regulation and *Salmonella* SiiE-mediated adhesion, but not extra- and intracellular *Salmonella* growth. However, the inhibitory activity of EcN against *Salmonella* invasion always correlated with EcN adhesion capacities. EcN mutants defective in the expression of F1C fimbriae and flagellae were less adherent and less inhibitory toward *Salmonella* invasion. Another *E. coli* strain expressing F1C fimbriae was also adherent to IPEC-J2 cells, and was similarly inhibitory against *Salmonella* invasion like EcN.

**Conclusions:**

We propose that EcN affects *Salmonella* adhesion through secretory components. This mechanism appears to be common to many *E. coli* strains, with strong adherence being a prerequisite for an effective reduction of SiiE-mediated *Salmonella* adhesion.

## Introduction

*E. coli* Nissle 1917 (EcN; Mutaflor) is a widely employed probiotic strain and several *in vivo* studies demonstrated its promising probiotic activity in humans and animals [1], [2], [3], [4], [5], [6]. Proposed probiotic actions of EcN include effects on pathogens, host epithelial cells, host smooth muscle cell activity and the host immune system [7], [8], [9], [10], [11], [12], [13]. *In vitro*, EcN has been shown to prevent invasion of host cells by several pathogens, including *Salmonella*, *Yersinia*, *Shigella*, *Legionella*, *Listeria* and adherent-invasive *E. coli* [14], [15], [16]. These studies demonstrated that EcN inhibited invasion in a dose-dependent manner. Interestingly, EcN supernatants were also effective in inhibiting invasion. However, the underlying molecular mechanisms involved in this process are poorly understood to date.

Successful probiotic action of a bacterial strain is often associated with its colonization of the intestine. The colonization of hosts by EcN can be very successful, but is likely specific for each individual [15], [17]. EcN has been shown to express type 1 fimbriae and F1C fimbriae (which have usually been associated with uropathogenic *E. coli*), but not P and S fimbriae [18]. Recently, it was demonstrated that EcN F1C fimbriae or cellulose production play an important role in EcN biofilm formation, adherence to intestinal epithelial cells *in vitro,* and intestinal colonization of mice [19], [20].

The aim of the present study was to characterize the effects of EcN on *Salmonella* invasion of the porcine intestinal epithelial cell line IPEC-J2 [21], with a focus on EcN effects on single *Salmonella* infection steps. In addition, EcN adhesion capacities were tested as possible requisites for an EcN specific probiotic activity. We show that adhesion rates always correlate with inhibitory effects against *Salmonella* invasion, and the initial *Salmonella* invasion process, especially adhesion, is likely affected.

## Materials and Methods

### Bacterial strains used in this study

*E. coli* Nissle 1917 DSM6601 (O6:H1:K5, EcN) was kindly provided by G. Breves (Hannover, Germany). *E. coli* MG1655 was kindly provided by C.A. Gross (San Francisco, USA). *Salmonella enterica* serovar Typhimurium (*Salmonella Typhimurium*) strain SL1344 was kindly provided by F. Norel (Paris, France). SL1344*::kan* was generated by introduction of a kanamycin resistance cassette into the downstream, non-coding region between the *avrA* and *sitD* genes of *Salmonella typhimurium* LT2 as previously described [22]. Following PCR screening for correct insertion and orientation, the kanamycin resistance cassette was introduced into strain SL1344 by bacteriophage P22 transduction using standard protocols. The non-invasive *Salmonella Typhimurium* strain VV341 (SL1344 *hilA*-339::*kan*) was kindly provided by C.A. Lee (Boston, USA). The non-invasive *Salmonella Typhimurium* strain SB161 (SL1344 *invG*) was kindly provided by M. Hensel (Erlangen, Germany) and was transformed with the pEGFP plasmid. β-Galactosidase assays were performed with strains CL87 (SL1344 *hilA*(*iagB*)::*lacZY*), EE635 (SL1344 *hilC9*::Tn5*lacZY*), and RL696 (SL1344 *hilD696*::*lacZY*) provided by C.A. Lee. Strain KT4166 harboring a *lacZ* fusion to the SPI4-encoded *siiE* gene (SL1344 *icgA1*(*siiE*)::*MudJ*(*kan*)) was constructed by P22 transduction of the *siiE*::*MudJ*(*kan*) fusion into strain SL1344 with kanamycin selection using lysates prepared on strain JS296 (J.M. Slauch, Urbana, USA).

*E. coli* 140815 (IMT13962) was isolated from feces of a clinically healthy pig and found by PCR to be negative for the virulence genes *eae*, *stx2e*, *faeG*, *fanA*, *fasA*, *fedA*, *fimF41a*, *est-1a, eltB-Ip* [15]. A Δ*fim* mutant of EcN was provided by T. Ölschläger [14]. Additional Δ*focA* and Δ*fliA* mutants of EcN were generated in this study using the protocol of Datsenko and Wanner [22]. Both mutants were complemented with the plasmid pACYC177 harboring sequences encoding *focA* or *fliA* of *E. coli* CFT073. This was necessary as there were no gene sequences from EcN for *focA* and *fliA* available, but the genome of EcN shows high homology to uropathogenic *E. coli* CFT073 [18]. *E. coli* WS15C1, WS30C1 and WS46C1 were isolated from intestinal contents of wild boars ([23] and this study).

### *In vitro* assays

#### IPEC-J2 cell culture conditions

The porcine intestinal epithelial cell line IPEC-J2 [21] was grown in Dulbecco's modified Eagle Medium (DMEM) HAM'S/F-12 (1∶1) (Biochrom, Germany), supplemented with 5% fetal calf serum, and maintained in an atmosphere of 5% CO_2_ at 37°C. Cells reached confluence after 3–4 days and were used consistently within 8 days from seeding. Cell cultures were tested routinely and found to be free from mycoplasma contamination.

### *In vitro Salmonella* invasion assays

Invasion assays were performed essentially as previously described [24]. *E. coli* were grown in LB medium to an optical density at 600 nm (OD_600_) of approximately 1, washed by centrifugation, re-suspended in cell culture medium and adjusted by dilution to provide a multiplicity of infection (MOI) of 100∶1 or 10∶1 *E. coli* to host cells in culture wells of a 24-well plate using a conversion of approximately 3×10^8^ bacteria/ml/OD_600_. Confluent monolayers of IPEC-J2 cells were first incubated with the respective *E. coli* strain for 2 or 6 hours at 37°C. Cells were washed three times with PBS to remove non-adherent *E. coli*. *S. Typhimurium* was grown in LB medium to an OD_600_ of approximately 2, washed by centrifugation, re-suspended in cell culture medium and adjusted by dilution to provide a MOI of 100∶1 or 1∶1 *Salmonella* to host cells using a conversion of approximately 3×10^8^ bacteria/ml/OD_600_. Confluent monolayers were infected after *E. coli* pre-incubation for one hour, followed by an additional hour of incubation in media containing 50 µg/ml gentamicin to kill extracellular bacteria. Infected cells were washed twice with PBS and lysed with 0.1% Triton X-100 in deionized, distilled water. Dilutions of the resulting cell lysates were plated on LB agar plates for determination of intracellular bacterial counts.

For kinetics of intracellular *Salmonella* growth, IPEC-J2 cells were infected with *Salmonella Typhimurium* at a MOI of 1∶1 for one hour, with an additional incubation of one hour in media containing 50 µg/ml gentamicin to kill extracellular bacteria (time point “2 hours”). IPEC-J2 cells were washed three times with media and incubated with the respective *E. coli* strain for 2 hours with a MOI of 100∶1 *E. coli* to host cells, followed by incubation in media containing 50 µg/ml gentamicin for one hour to kill extracellular bacteria (time point “5 hours”). Finally, cells were incubated over 19 hours in media containing 10 µg/ml gentamicin (end time point “24 hours”).

To determine the effects of EcN in mixed *E. coli* cultures, EcN was mixed with other *E. coli* in a ratio of 1∶1 (mixture with one other *E. coli* strain) or in a ratio of 1∶1∶1 (mixture with two other *E. coli* strains) with a final MOI in the mixture of 100∶1 *E. coli* to host cells.

To determine the effects of *E. coli* supernatants, *E. coli* were grown in DMEM HAM'S/F-12 (1∶1) supplemented with 5% fetal calf serum at 37°C to an OD_600_ of 1. Bacteria were centrifuged at 800×g for 5 min. Supernatants were sterile-filtered (pore size 0.22 µm) and used in the cell culture assays.

To determine the effects of *E. coli* supernatants on *Salmonella* growth rates, *Salmonella* counts were determined in the culture supernatants during the respective invasion assays. After a one-hour incubation step with *Salmonella*, the supernatant of each well was removed and plated in serial dilutions on LB agar plates. Invasion and adhesion assays were performed in duplicate.

### *In vitro Salmonella* adhesion assays

*Salmonella* adhesion assays with SL1344 *hilA*-339::*kan* were performed similarly to the invasion assays. *E. coli* were grown in LB medium to an OD_600_ of approximately 1, washed by centrifugation, re-suspended in cell culture medium and adjusted by dilution to provide a MOI of 100∶1 *E. coli* to host cells in culture wells of a 24-well plate. Confluent monolayers of IPEC-J2 cells were first incubated with the respective *E. coli* strain for 2 or 6 hours at 37°C. Cells were washed three times with PBS to remove non-adherent *E. coli*. SL1344 *hilA*-339::*kan* was grown in LB medium to an OD_600_ of approximately 2, washed by centrifugation, re-suspended in cell culture medium and adjusted by dilution to provide a MOI of 100∶1 *Salmonella* to host cells. Confluent monolayers were infected after *E. coli* pre-incubation for one hour. After the one hour incubation of IPEC-J2 cells with SL1344 *hilA*-339::*kan*, cells were washed 3 times with PBS to remove non-adherent *Salmonella*. IPEC-J2 cells were lysed and lysates were plated on to LB agar plates containing 50 µg/ml kanamycin for determination of adherent *Salmonella* counts.

*Salmonella* adhesion assays with SL1344 pEGFP *invG*-339::*kan* were performed similarly to the *Salmonella* adhesion assays with SL1344 *hilA*-339::*kan* until the washing step after the 1 hour incubation with *Salmonella*. After washing IPEC-J2 cells 3 times with PBS to remove non-adherent *Salmonella,* cell culture plates were analyzed by the Aklides apparatus (GA Generic Assays GmbH, Germany). This apparatus automatically recognizes fluorescent *Salmonella* cells, photographs cell monolayers with adherent fluorescent bacteria, and analyses pictures by counting fluorescent cell numbers. IPEC-J2 cell lysis, dilutions of lysates, plating of dilutions and counting of bacterial colonies are omitted.

### *In vitro E. coli* adhesion assays

*E. coli* were grown in LB medium to an OD_600_ of approximately 1, washed by centrifugation, re-suspended in cell culture medium and adjusted by dilution to provide a MOI of 100∶1 *E. coli* to host cells in culture wells of a 24-well plate. Confluent monolayers of IPEC-J2 cells were incubated with the respective *E. coli* strain (EcN, EcN mutants, *E. coli* WS15C1, *E. coli* WS30C1 and *E. coli* WS46C1) for 2 hours at 37°C. Cells were washed three times with PBS to remove non-adherent *E. coli*. IPEC-J2 cells were lysed and lysates were plated on to LB agar plates for determination of adherent *E. coli* counts.

### β-Galactosidase assays

β-Galactosidase assays were performed as previously described [25], [26] with *Salmonella* strains grown aerobically to late-log/early stationary phase (OD_600_ ∼2), unless otherwise noted.

### Statistical analysis

*P* values were calculated using the Student's t test implemented in the Statistical Package for the Social Sciences (SPSS Statistics; version 17.0).

## Results

### *E. coli* Nissle 1917 as well as *E. coli* supernatants inhibit *Salmonella* invasion into IPEC-J2 cells

**Figure 1:**
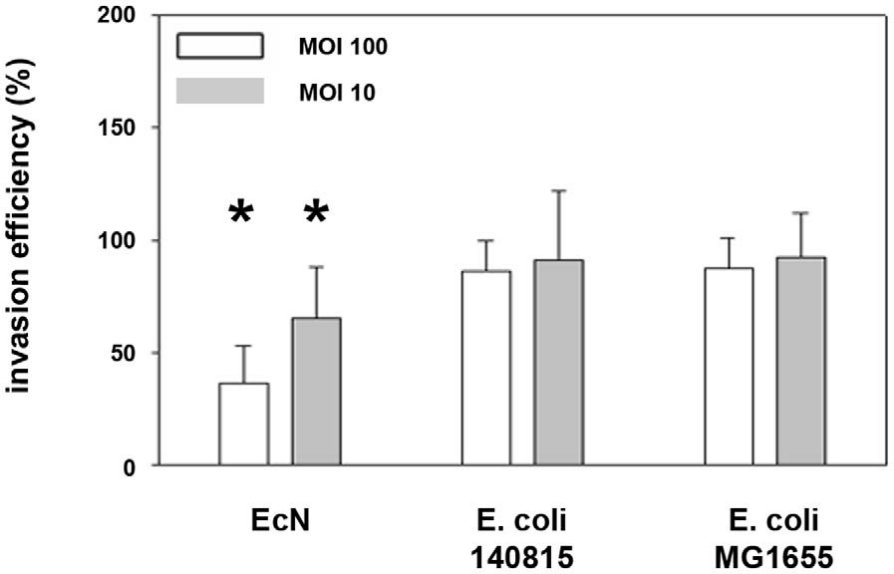
Invasion efficiency of *Salmonella Typhimurium* into IPEC-J2 cells after pre-incubation with *E. coli* Nissle 1917. Confluent monolayers of IPEC-J2 cells were pre-incubated with *E. coli* Nissle 1917 (EcN), *E. coli* 140815 or *E. coli* MG1655 at an MOI of 100∶1 or 10∶1 bacteria to host cells. After two hours, cells were washed and infected with *Salmonella Typhimurium* using an MOI of 100∶1 *Salmonella* to host cells. Invasion levels in percent (%) are expressed as invasion of *Salmonella* relative to invasion without pre-incubation with *E. coli* (*Salmonella* mono-infection). The data are the mean ± S.E.M. of at least three separate experiments in duplicate wells. * = p<0.01 compared to *Salmonella* mono-infection.

**Figure 2:**
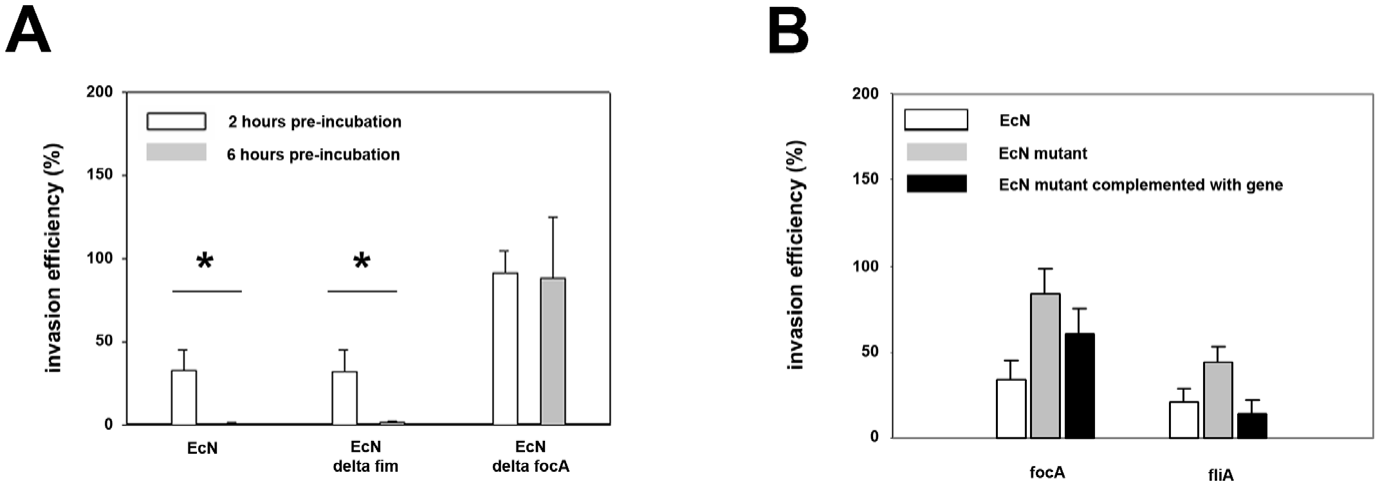
Inhibitory effects of *E. coli* Nissle 1917 on *Salmonella Typhimurium* invasion is dependent on adhesion. Confluent monolayers of IPEC-J2 cells were pre-incubated with *E. coli* Nissle 1917 (EcN), EcN Δ*focA*, EcN Δ*fim* or EcN Δ*fliA* using an MOI of 100∶1 *E. coli* to host cells. After two or six hours, cells were washed and infected with *Salmonella Typhimurium* using an MOI of 100∶1 *Salmonella* to host cells. Invasion levels in percent (%) are expressed as invasion of *Salmonella* relative to invasion without pre-incubation with *E. coli* (*Salmonella* mono-infection). The data are the mean ± S.E.M. of at least three separate experiments in duplicate wells. * = p<0.01 compared to *Salmonella* mono-infection. A) Effects of EcN Δ*focA* and EcN Δ*fim* mutants on *Salmonella* invasion after a 2 or 6 hours pre-incubation period. B) Effects of EcN Δ*focA* and EcN Δ*fliA* mutants and their respective strains complemented with the plasmid pACYC 177 containing the relevant gene on *Salmonella* invasion after 2 hours pre-incubation.

Initially, we verified a probiotic effect of *E. coli* Nissle 1917 (EcN) on *Salmonella* invasion of porcine intestinal epithelial cells (IPEC-J2). We compared the effects of EcN with those of two control *E. coli* strains (*E. coli* 140815 and *E. coli* MG1655), and the effects of EcN in a mix with these two strains, and included two multiplicities of infections (MOI). In general, employing the gentamicin protection assay the invasion efficiency of *Salmonella Typhimurium* strain SL1344 into the porcine intestinal epithelial cell line IPEC-J2 was 27%. A two-hour pre-incubation of IPEC-J2 cells with EcN resulted in a decrease in *Salmonella* invasion efficiency, while pre-incubation with *E. coli* 140815 or *E. coli* MG1655 did not. This effect was stronger using an MOI of 100∶1 (*E. coli*:epithelial cells) compared to 10∶1 (Figure 1). The inhibitory effect of EcN was markedly increased by a pre-incubation period of six hours compared to two hours (Figure 2A).

**Figure 3:**
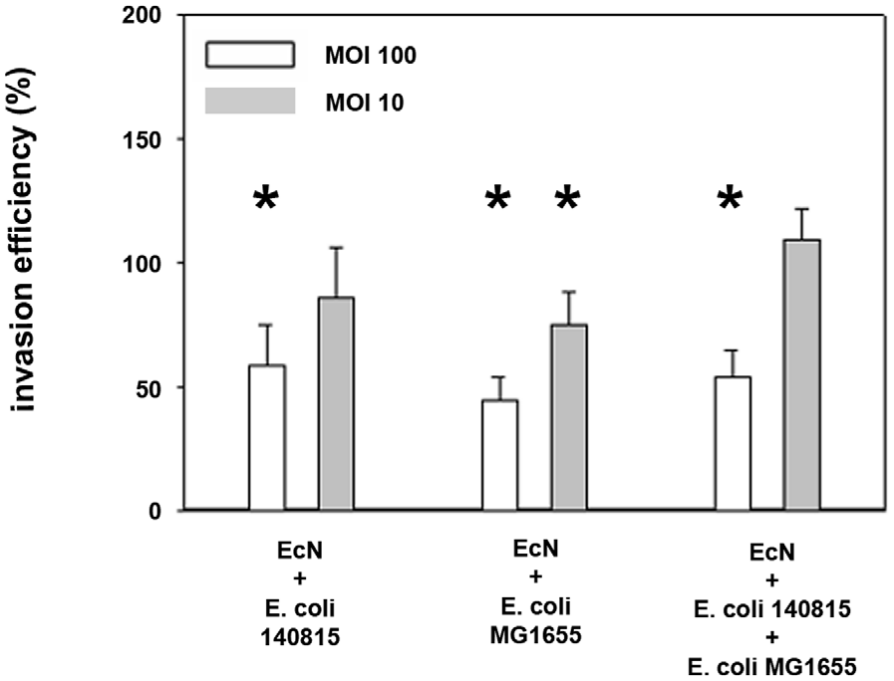
Invasion efficiency of *Salmonella Typhimurium* into IPEC-J2 cells after pre-incubation with *E. coli* mixed cultures. Confluent monolayers of IPEC-J2 cells were pre-incubated with *E. coli* Nissle 1917 (EcN) monocultures or mixed cultures using an MOI of 100∶1 or 10∶1 *E. coli* to host cells. After two hours, cells were washed and infected with *Salmonella Typhimurium* using an MOI of 100∶1 *Salmonella* to host cells. Invasion levels in percent (%) are expressed as invasion of *Salmonella* relative to invasion without pre-incubation with *E. coli* (*Salmonella* mono-infection). The data are the mean ± S.E.M. of at least three separate experiments in duplicate wells. * = p<0.01 compared to *Salmonella* mono-infection.

Inhibition of *Salmonella* invasion by EcN was also observed in mixed *E. coli* cultures although the effects on invasion were less effective in mixed *E. coli* cultures compared to EcN in the monoculture model. Using a mixture of EcN:*E. coli* 140815:*E. coli* MG1655 (1∶1∶1), at a total MOI of 10∶1 the inhibitory effects of EcN were abolished (Figure 3).

**Figure 4:**
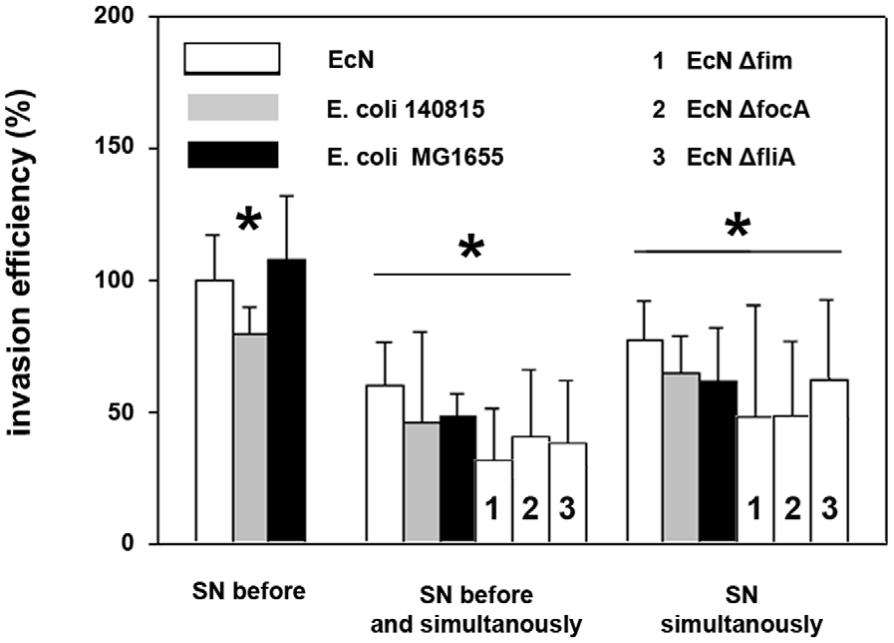
Invasion efficiency of *Salmonella Typhimurium* into IPEC-J2 cells after incubation with *E. coli* culture supernatants. Confluent monolayers of IPEC-J2 cells were pre-incubated (SN before) and/or co-incubated (SN simultaneously) with *E. coli* supernatants (SN). Cells were infected with *Salmonella Typhimurium* using an MOI of 100∶1 *Salmonella* to host cells. Invasion levels in percent (%) are expressed as invasion of *Salmonella* relative to invasion without pre- and/or co-incubation with *E. coli* SN. The data are the mean ± S.E.M. of at least three separate experiments in duplicate wells. * = p<0.05 compared to *Salmonella* infection without influence of *E. coli* SN. EcN: *E. coli* Nissle 1917.

We also tested the effects of cell-free EcN supernatants compared to supernatants of the two control strains. To create a scenario relevant to the initial experiments with bacteria (for which EcN was present in the pre- and co-incubation period), we included pre- and pre-/co-incubation experiments using *E. coli* culture supernatants. As shown in Figure 4, pre-incubation with *E. coli* supernatants (*E. coli* 140815) showed little or no effects on *Salmonella* invasion efficiency, while pre-/co-incubation noticeably inhibited *Salmonella* invasion. We tested the hypothesis that co-incubation of *E. coli* supernatants with *Salmonella* was predominantly responsible for the inhibitory effect in subsequent co-incubation experiments. However, co-incubation of supernatants of all three tested *E. coli* strains similarly inhibited *Salmonella* invasion (Figure 4) as was the case with *E. coli* pre-incubations. Also supernatants of EcN mutants Δ*focA* (F1C fimbriae), Δ*fimA* (type 1 fimbriae) and Δ*fliA* inhibited *Salmonella* invasion comparable to EcN wildtype (Figure 4).

### Effects of *E. coli* supernatants on extracellular growth

**Figure 5:**
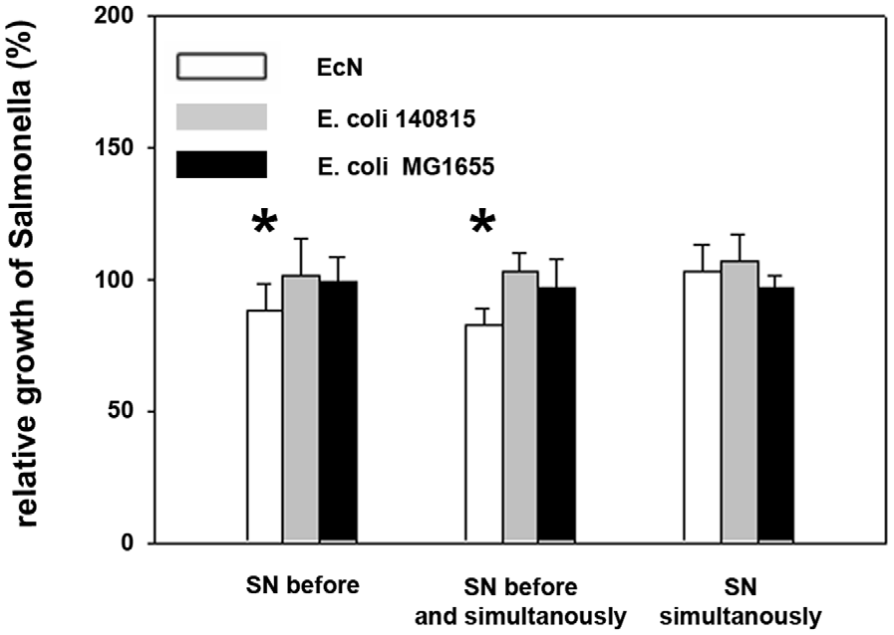
Effects of *E. coli* supernatants on growth of *Salmonella Typhimurium*. Confluent monolayers of IPEC-J2 cells were pre- and/or co-incubated with *E. coli* supernatants (SN). Cells were infected with *Salmonella Typhimurium* using an MOI of 100∶1 *Salmonella* to host cells for one hour. Thereafter, numbers of extracellular, non-adherent *Salmonella* were determined. Growth rates are expressed as growth in percent (%) relative to *Salmonella* growth in cell culture medium (*Salmonella* mono-infection). The data are the mean ± S.E.M. of at least three separate experiments in duplicate wells. * = p<0.01 compared to *Salmonella* mono-infection. EcN: *E. coli* Nissle 1917.

The inhibitory effect of EcN against *Salmonella* invasion might have been due to the inhibition of *Salmonella* growth by *E. coli* supernatants, which inevitably affects invasion efficiencies. To exclude such effects, *Salmonella* numbers were determined in cell culture supernatants in parallel to the invasion assays. Supernatants of all three *E. coli* did not, or only slightly (EcN), affect extracellular growth of *Salmonella* within the one hour *Salmonella* incubation time (Figure 5).

### Effects of *E. coli* on adhesion of *Salmonella*

**Figure 6:**
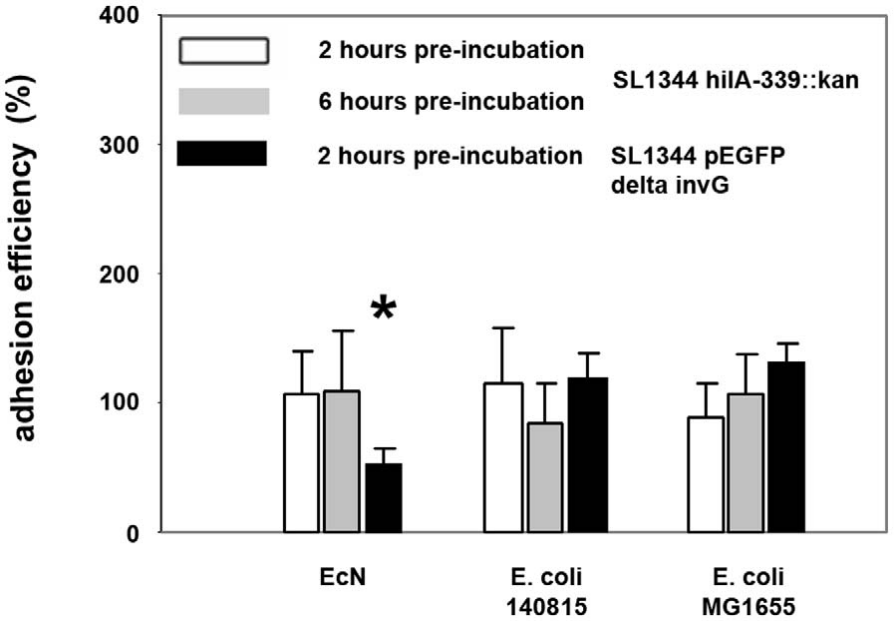
Adhesion efficiency of *Salmonella Typhimurium* to IPEC-J2 cells after pre-incubation with *E. coli*. Confluent monolayers of IPEC-J2 cells were pre-incubated with *E. coli* Nissle 1917 (EcN), *E. coli* 140815 or *E. coli* MG1655 using an MOI of 100∶1 *E. coli* to host cells. After two or six hours, cells were washed and infected with non-invasive *Salmonella Typhimurium* SL1344 *hilA*-339::*kan* or SL1344 pEGFP *invG*-339::*kan* using an MOI of 100∶1 *Salmonella* to host cells. Adhesion levels in percent (%) are expressed as adhesion of *Salmonella* relative to adhesion without pre-incubation with *E. coli* (*Salmonella* mono-infection). The data are the mean ± S.E.M. of at least three separate experiments in duplicate wells. * = p<0.01 compared to *Salmonella* mono-infection.

We determined whether EcN inhibited *Salmonella* adhesion, which is also a prerequisite for *Salmonella* invasion. *Salmonella* adhesion assays were performed with non-invasive *S. Typhimurium* SL1344 *hilA*-339::*kan*. All three *E. coli* strains showed no effects on *Salmonella* adhesion to IPEC-J2 cells in pre-incubation experiments (Figure 6). Even after a six-hour pre-incubation period with *E. coli*, adhesion of *S. Typhimurium* SL1344 *hilA*-339::*kan* was not affected (Figure 6).

Additional *Salmonella* adhesion tests were performed with non-invasive *S. Typhimurium* SL1344 pEGFP *invG*-339::*kan*. This mutant adhered twice to IPEC-J2 cells than non-invasive *S. Typhimurium* SL1344 *hilA*-339::*kan*. EcN inhibited SL1344 pEGFP *invG*-339::*kan* adhesion by 40%. In contrast, *E. coli* strain MG1655 and 140815 enhanced *Salmonella* adhesion to IPEC-J2 cells in pre-incubation experiments (Figure 6). The kanamycin resistance of non-invasive *Salmonella* mutants does not contribute to this effect since strain SL1344*::kan* harboring a chromosomal kanamycin resistance cassette in a non-coding, intergenic region not affecting the expression of any genes was similarly invasive and had a similar probiotic effect against *Salmonella* invasion (reduction of invasion by 55.3%) as the wild type strain itself.

### *E. coli* Nissle 1917 does not inhibit intracellular growth of *Salmonella*

**Figure 7:**
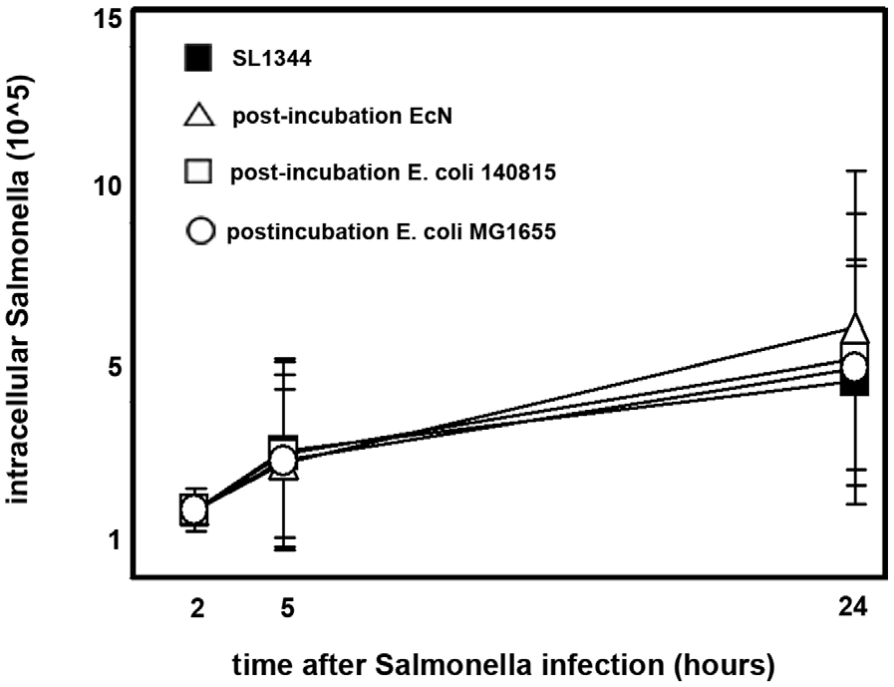
Intracellular growth of *Salmonella Typhimurium* in IPEC-J2 cells after post-incubation with *E. coli* Nissle 1917. Confluent monolayers of IPEC-J2 cells were infected with *Salmonella Typhimurium* for one hour using an MOI of 1∶1 *Salmonella* to host cells. After one additional hour of incubation in media containing gentamicin, IPEC-J2 cells were incubated with *E. coli* Nissle 1917 (EcN), *E. coli* 140815 or *E. coli* MG1655 for two hours using an MOI of 100∶1 *E. coli* to host cells, followed by incubation in media with gentamicin. Intracellular *Salmonella* numbers are presented per well of a 24-well plate. The data are the mean ± S.E.M. of at least three separate experiments in duplicate wells.

To test whether EcN might have been able to affect *Salmonella* post-invasion, EcN and the two control strains were incubated with *Salmonella* in post-incubation experiments. Here, IPEC-J2 cells were initially infected with *Salmonella* for one hour, and extracellular *Salmonella* were inactivated by incubation with gentamicin for another hour. After removal of gentamicin IPEC-J2 cells with intracellular *Salmonella* were incubated with *E. coli* for 2 hours and then incubated for up to 20 hours with medium containing gentamicin to kill extracellular bacteria. Intracellular *Salmonella* were determined at 2, 5 and 24 hours after the initial *Salmonella* incubation period. As shown in Figure 7, all three *E. coli* had no effect on intracellular *Salmonella* growth at 2, 5 as well as 24 hours after the initial *Salmonella* incubation.

### F1C fimbriae mediates inhibitory effects of EcN on *Salmonella* invasion

Adhesion might be a prerequisite for the inhibitory effect of EcN. To test this, we used EcN Δ*focA* (F1C fimbriae), Δ*fimA* (type 1 fimbriae) and Δ*fliA* (flagellae) mutants. Adhesion by strains EcN Δ*focA* (reduction by 92.7%) and EcN Δ*fliA* (reduction by 47.9%) on IPEC-J2 cells was reduced compared to EcN wild type strain. After complementation with the pACYC 177 plasmids, respectively containing the *focA* or *fliA* gene, adhesion was enhanced to 53.4% (focA) and 122.1% (fliA), compared to the EcN wild type strain. Adhesion by strain EcN Δ*fimA* on IPEC-J2 cells was not reduced compared to EcN wild type strain. A decrease or increase in adhesion correlated with a decrease or increase of the inhibitory effect on *Salmonella* invasion, respectively. Thus EcN Δ*focA* did not inhibit *Salmonella* invasion compared to EcN wildtype strain, whereas EcN Δ*fliA* inhibited *Salmonella* invasion by 50%. EcN Δ*fimA* inhibited *Salmonella* invasion at levels similar to those of EcN wildtype strain (Figure 2). The results were more prominent using a 6-hour EcN pre-incubation period.

**Figure 8:**
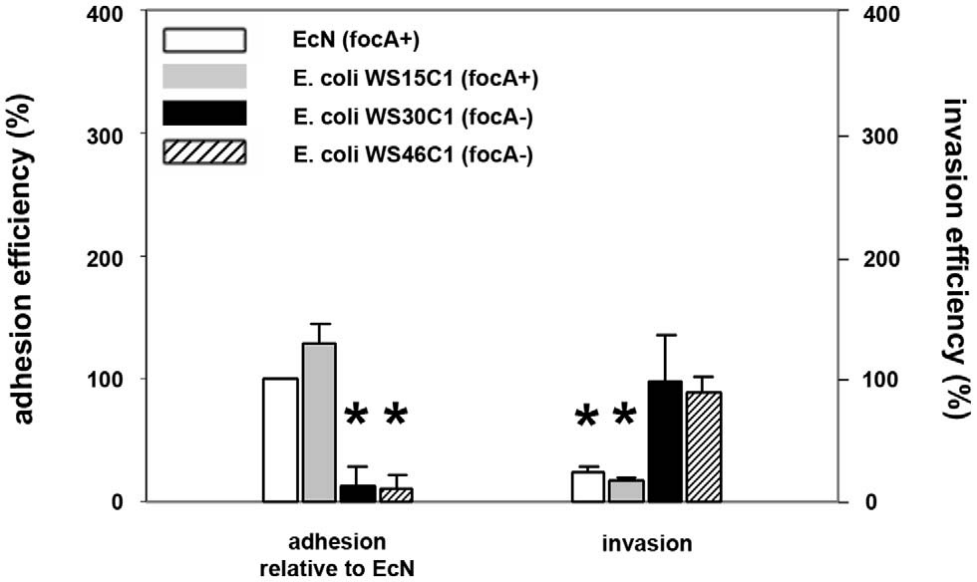
Inhibitory effects of *focA*-positive and *focA*-negative *E. coli* isolates on *Salmonella Typhimurium* invasion. Confluent monolayers of IPEC-J2 cells were pre-incubated with *E. coli* Nissle 1917 (EcN, *focA*-positive strain), *E. coli* WS15C1 (*focA*-positive strain), *E. coli* WS30C1 (*focA*-negative strain) and *E. coli* WS46C1 (*focA*-negative strain) using an MOI of 100∶1 *E. coli* to host cells. After two hours, cells were washed and adhesion efficiencies of *E. coli* isolates were determined (left side); alternatively, cells were washed after two hours and infected with *Salmonella Typhimurium* using an MOI of 100∶1 *Salmonella* to host cells (right side). Adhesion levels in percent (%) were expressed relative to adhesion of EcN. Invasion levels in percent (%) were expressed relative to *Salmonella* invasion without pre-incubation with bacteria (*Salmonella* mono-infection). The data are the mean ± S.E.M. of at least three separate experiments in duplicate wells. * = p<0.01 compared to EcN adhesion (left side) or *Salmonella* mono-infection (right side).

To test whether adhesion via F1C fimbriae is essential for a subsequent inhibitory effect of EcN and the specificity of EcN inhibition, we compared adhesion rates as well as inhibition between EcN and another *focA* gene-positive (WS15C1), and two other *focA* gene-negative (WS30C1 and WS46C1) *E. coli* strains. These bacteria all carry the type 1 fimbriae and flagellae and were isolated from the intestine of clinically healthy wild boars [23]. As shown in Figure 8, adhesion rates of the *focA* gene-positive strain WS15C1 were higher compared to EcN, but *focA* gene-negative strains WS30C1 and WS46C1 adhered much less compared to EcN. Additionally, these high adhesion rates correlated with high inhibitory effects of EcN and WS15C1 on *Salmonella* invasion (Figure 8).

### Effects of EcN on expression of *Salmonella* invasion genes

**Figure 9:**
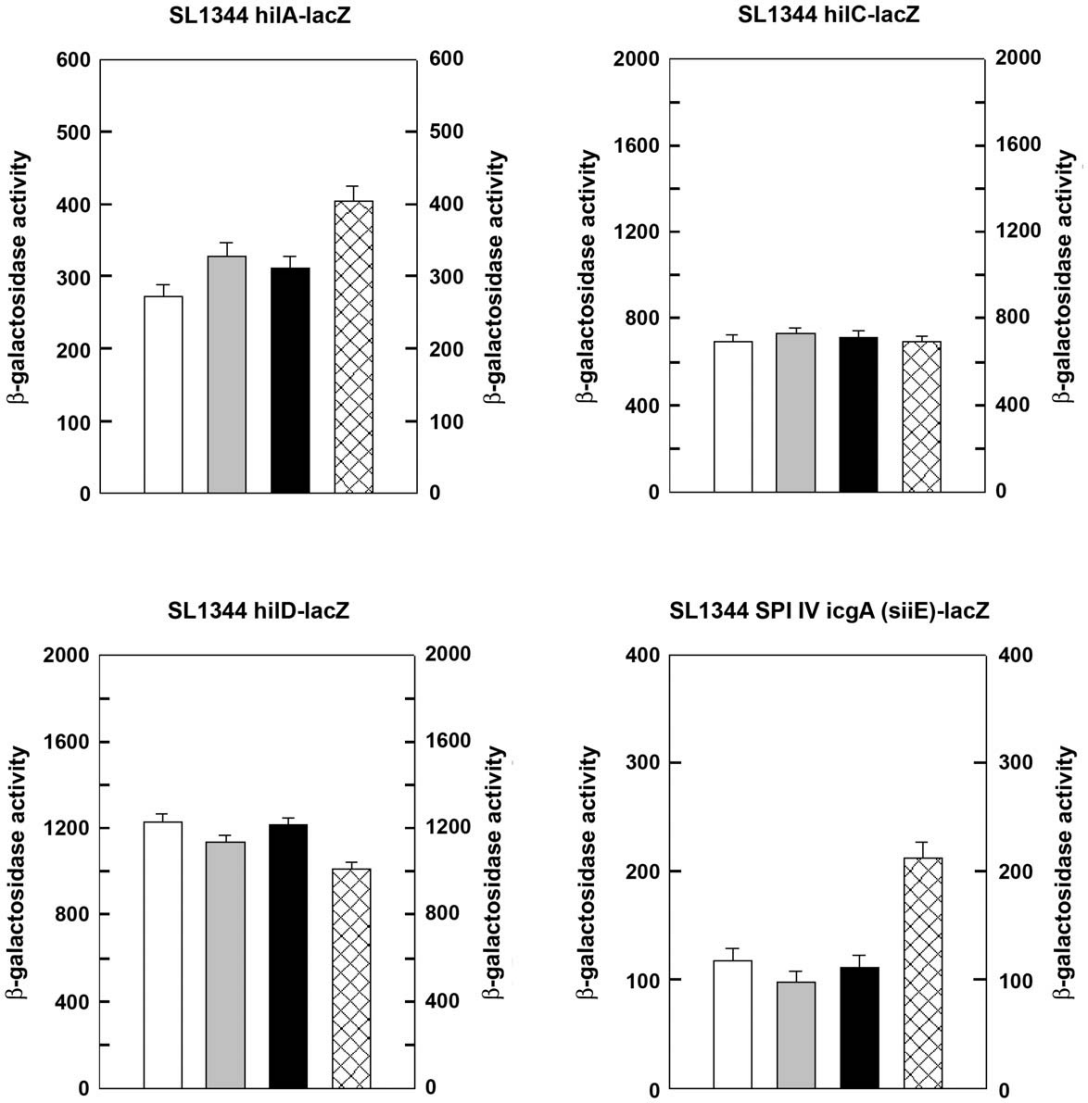
*Salmonella* invasion gene regulation by *E. coli* supernatants. *E. coli* were cultivated in cell culture medium (DMEM HAM'S/F-12) until an OD_600nm_ = 1. Supernatants were collected by centrifugation with subsequent sterile filtration. Subsequently, SL1344 fusion strains (SL1344 *hilC*-*lacZ*, SL1344 *hilD*-*lacZ*, SL1344 *hilA*-*lacZ*, SL1344 *icgA*(*siiE*)-*lacZ*) were cultivated in supernatants of EcN, *E. coli* 140815 or *E. coli* MG1655. B-Galactosidase activity was measured as previously described [25], [26]. The results shown are representative of at least two independent experiments. White bar: *Salmonella* grown in EcN supernatant, gray bar: *Salmonella* grown in *E. coli* 140815 supernatant, black bar: *Salmonella* grown in *E. coli* MG1655 supernatant, patterned bar: *Salmonella* grown in pure cell culture medium.

As the previous results indicated that *Salmonella* adhesion - but not extracellular or intracellular *Salmonella* growth - was affected by EcN, we tested the effects of EcN supernatants on the expression of *Salmonella* invasion gene regulatory and dependent genes. *Salmonella* strains harboring *lacZ* fusions to the invasion locus regulatory genes *hilC*, *hilD*, *hilA*, and the SPI4-encoded *siiE* gene were incubated with supernatants of EcN, *E. coli* 140815 or *E. coli* MG1655. As shown in Figure 9, expression of HilC was not affected by any of the three *E. coli* supernatants. HilD expression was slightly enhanced by all three *E. coli* supernatants and HilA and SiiE expressions were inhibited by all three *E. coli* supernatants.

## Discussion

Probiotic *E. coli* Nissle 1917 (EcN) is being successfully used for the prevention and treatment of various intestinal diseases of humans and animals. However, the basis of its mode of action remains largely unanswered. EcN has been shown in *in vitro* studies to protect human embryonic intestinal epithelial cells (INT407 cells) against infection by different enteropathogens, including enteroinvasive bacteria, but the underlying mechanisms were not clarified [14], [16]. In the present study, we initially defined possible probiotic effects of EcN against *Salmonella* invasion of porcine intestinal epithelial cells (IPEC-J2) and subsequently verified such probiotic effects against single stages of the *Salmonella* invasion process.

EcN successfully inhibited *Salmonella* infection of IPEC-J2 cells. The specificity of this effect was demonstrated in that two control *E. coli* strains, 140815 and MG1655, showed no such inhibitory effects. Inhibition of *Salmonella* infection could have been due to effects on several infection steps including inhibition of intracellular and extracellular *Salmonella* growth, inhibition of adhesion, or other factors. The probiotic effect of EcN was found to be highly dependent upon its adherence to IPEC-J2 cells, preferentially through F1C fimbriae (Figure 2). *Salmonella* adhesion and *Salmonella* adhesion gene expressions were affected by EcN and/or EcN supernatants respectively, but not *Salmonella* extracellular or intracellular growth, supporting a role of adhesion genes in the probiotic effects.

We propose two mechanisms for the probiotic effect of EcN. First, EcN supernatants could be responsible for this effect. This might be mediated through interactions with the *Salmonella* SiiE-dependent adhesion mechanism, as well as down-regulation of the expression of the adhesion factor SiiE. We suggest that *Salmonella* adhesion was reduced via SiiE-mediated adhesion by EcN since adhesion of a non-invasive *invG Salmonella* mutant with a functional SiiE mediation system was affected, whereas adhesion of a non-invasive *Salmonella* mutant defective in *hilA* with an SiiE-minus genotype was not, however; *E. coli* supernatants suppressed both SiiE and HilA expression, as well as suppression of both genes contributed to the probiotic effect. HilA is a transcriptional regulator of the OmpR/ToxR family that is encoded on *Salmonella* pathogenicity island 1 and plays a key role in the regulation of invasiveness of *Salmonella Typhimurium*. HilA has been shown to be required for the optimal expression of both invasion genes as well as the adhesin SiiE [27], [28]. While HilA-dependent regulation has been intensively studied, other HilA-dependent adhesion genes have not been reported so far. HilA expression is also regulated by the regulators HilC and HilD [29]. In our study, HilC expression was not influenced by *E. coli* supernatants, while HilD expression was slightly enhanced. Thus the regulation of HilA by HilD, which would have up-regulated HilA and SiiE expression, was weaker than the direct effects of *E. coli* supernatants on HilA. SiiE is secreted from *Salmonella* by a type I secretion system into the cell culture supernatant and can function as an adhesin when in contact with polarized epithelial cells. The IPEC-J2 cell line has also been shown to form a polarized cell monolayer [21], [30]. This mechanism might not be EcN-specific since supernatants from other *E. coli* also similarly inhibited *Salmonella* invasion, and similarly regulated *Salmonella* virulence gene expression.

A second mechanism is suggested by the strong adherence of EcN on IPEC-J2 cells and the subsequent inhibition of *Salmonella* invasion which may be a major contributing factor in the probiotic effect of EcN. This strong adhesion was EcN-specific compared to the control strains 140815 and MG1655 and was predominantly mediated by F1C fimbriae. However, other F1C fimbriae expressing *E. coli* strains could also strongly adhere to IPEC-J2 cells and reduce *Salmonella* invasion, as was the case with *E. coli* strain WS15C1.

In summary, in the presence of *E. coli*, components present in culture supernatants appear to reduce *Salmonella* SiiE-mediated adhesion by down-regulation of SiiE production. *E. coli* supernatant compounds might also block SiiE-mediated adhesion by binding to either the SiiE protein itself or to SiiE receptors on the host cell surface. Such effects on the adhesion mediated by the SiiE protein are a subject for future research. In the presence of high concentrations of *E. coli* supernatants components might bind to the host cell surface. After washing host cells, followed by incubation in fresh cell culture medium (pre-incubation experiments), these bound components might detach and be diluted by added fresh cell culture medium to concentrations that become ineffective in inhibiting *Salmonella* adhesion. If cells are however incubated at high concentrations of supernatants after washing (pre- plus post-incubation with supernatants), components of supernatants will still bind to the host cell surface in high concentrations and affect *Salmonella* adhesion. This might explain why pre- and co-incubation with *E. coli* supernatants resulted in a higher reduction of *Salmonella* invasion than pre-incubation or post-incubation only. Attachment to the cell surface through F1C fimbriae may support intimate EcN contact at the host cell surface and increase the local concentrations of supernatant compounds which decrease *Salmonella* adhesion and invasion.

A supernatant-dependent mechanism seems to be common for different *E. coli* strains (this study, [14]) as well as other bacterial species e.g. *Lactobacillus acidophilus*, *Lactobacillus casei*, *Lactobacillus plantarum*, *Pediococcus pentosaceus* and *Leuconostoc mesenteroides* [31], [32], [33]. In these latter studies, acidification of the medium followed by a bacteriocidal effect, the production of microcins, and other undefined bacteriocidal effects or inhibition of *Salmonella* growth were found to be responsible for probiotic supernatant-dependent mechanisms. However, in this study, *Salmonella* growth rates were similar in all different *E. coli* culture supernatants indicating that *E. coli* culture supernatants had no bacteriocidal effects against *Salmonella*. Such a finding does not rule out the possibility of other unknown supernatant factors binding to, and inhibiting SiiE or other molecules. Other authors reported that *L. casei* supernatant inhibited *Salmonella* invasion, but did not modify the viability of *Salmonella*. These authors supposed that a hypothetical substance of *L. casei* supernatant directly modified the ability of *Salmonella* to invade enterocyte-like cells *in vitro* in an acidic environment [33].

The adhesion of EcN to IPEC-J2 cells was a prerequisite for its probiotic effects. As shown in our study, F1C fimbriae as well as flagellae contributed to the adherence but not type 1 fimbriae. This is in accordance with recent observations that F1C fimbriae, not type 1 fimbriae, contributed to the adherence of EcN to the human larynx epithelial cell line Hep-2, and to their persistence in infant mouse colonization and biofilm formation [19]. EcN is therefore able to adhere via F1C fimbriae to different types of cells. The contribution of adherence to the probiotic effect was clearly indicated. For example, the adhesion of the EcN Δ*fliA* mutant complemented with a plasmid containing the *fliA* gene was enhanced to 122.1% compared to the EcN wild type strain, while *Salmonella* invasion decreased to 14.9% in the presence of the EcN Δ*fliA*/pACYC177 *fliA* construct, compared to 35% in the presence of the EcN wild type strain.

The conclusion of this study is three-fold. First, EcN suppressed *Salmonella* invasion by suppressing *Salmonella* adhesion. Second, effects of bacterial supernatants might be also common for other *E. coli*. And third, strong adherence is a prerequisite for the probiotic effect of EcN.
